# Services used by perinatal substance-users with child welfare involvement: a descriptive study

**DOI:** 10.1186/1477-7517-7-19

**Published:** 2010-08-31

**Authors:** Kenneth J McCann, Jean E Twomey, Donna Caldwell, Rosemary Soave, Lynne Andreozzi Fontaine, Barry M Lester

**Affiliations:** 1 Regional Child Protection Center, Blank Children's Hospital, Des Moines, IA 50309 USA; 2 Brown Center for the Study of Children at Risk, Women & Infants Hospital, Providence, RI, USA; 3 Brown Alpert Medical School, Providence, RI, USA; 4 National Perinatal Information Center, Providence, RI, USA; 5 Department of Psychology, Community College of Rhode Island, Warwick, RI, USA

## Abstract

**Background:**

Substance use during pregnancy often leads to involvement in the child welfare system, resulting in multiple social service systems and service providers working with families to achieve successful child welfare outcomes. The Vulnerable Infants Program of Rhode Island (VIP-RI) is a care coordination program developed to work with perinatal substance-users to optimize opportunities for reunification and promote permanency for substance-exposed infants. This paper describes services used by VIP-RI participants and child welfare outcomes.

**Methods:**

Data collected during the first four years of VIP-RI were used to identify characteristics of program participants, services received, and child welfare outcomes: closed child welfare cases, reunification with biological mothers and identified infant permanent placements.

**Descriptive Results:**

Medical and financial services were associated with positive child welfare outcomes. Medical services included family planning, pre- and post-natal care and HIV test counseling. Financial services included assistance with obtaining entitlement benefits and receiving tangible support such as food and clothing.

**Conclusions:**

Findings from this study suggest services that address basic family needs were related to positive child welfare outcomes. The provision of basic services, such as health care and financial assistance through entitlement benefits and tangible donations, may help to establish a foundation so mothers can concentrate on recovery and parenting skills. Identification of services for perinatal substance users that are associated with more successful child welfare outcomes has implications for the child welfare system, treatment providers, courts and families.

## Background

According to the Substance Abuse and Mental Health Service Administration (SAMHSA), 5% of pregnant women used illicit drugs in the past month [1]. The 2006 National Survey on Drug Use and Health found that rates of past month drug use were similar between non-pregnant women and recent mothers [2]. A study examining the prevalence of substance use among more than 7,800 pregnant women enrolled in prenatal care clinics identified 9% as using illicit substances when they were screened using the 4P's Plus tool, a measure comprised of four questions [3]. An endorsement of any of the questions is indicative of a positive screen.

Maternal substance use raises concerns about a woman's capability to adequately care for her child. Risk factors associated with substance abuse such as co-occuring psychiatric problems, violence, difficulties in interpersonal relationships, limited social support, unstable employment histories, and medical problems raise additional concerns about parenting abilities [4-7]. Estimates of the percentage of substance using parents involved in the child welfare system vary but there is general consensus that such families are disproportionately represented [7-11]. Maternal substance use is associated with greater numbers of infants entering the child welfare system [12-15].

When substance use during pregnancy results in child welfare involvement, multiple social service systems intervene to address the family's needs. There is limited information about specific services used by perinatal substance users with child welfare involvement. An examination of services received and child welfare outcomes can increase understanding about how best to intervene with perinatal substance users to achieve positive child welfare outcomes. The present study describes services received by women participating in the Vulnerable Infants Program of Rhode Island (VIP-RI) (described below) and three child welfare outcomes: 1) a closed child welfare case, 2) reunification with biological mother and 3) an identified permanent placement for the infant.

### Perinatal substance-users and services

The provision of comprehensive services is widely considered to improve treatment outcomes for pregnant and parenting substance-using women [16-18]. Women often can only engage in treatment when attention is given to pragmatic concerns, such as child care and transportation [19-22]. A review of 38 studies of substance abuse treatment for women found that more positive treatment outcomes were associated with the availiability of child care, prenatal care, mental health services, a focus on women's issues, women-only admissions and comprehensive treatment [19,23,24]. In another review, effective programs provided parent training and family interventions, visited in the home during the pregnancy to help prepare women for parenting responsibilities, and fostered collaboration among multiple agencies [25]. A policy review on early detection of prenatal substance exposure concluded that child developmental outcomes could be improved when child welfare systems collaborate with treatment providers and promote therapeutic as opposed to punitive actions [26].

Recent research has examined substance abuse treatment services and child welfare outcomes. A study investigating reunification of children whose mothers participated in the California Treatment Outcome Project (CalTOP), reported factors associated with reunification were mother's length of time in treatment and participation in programs that addressed larger family needs such as employment and education [9]. Mothers with more employment and psychiatric problems were less likely to achieve reunification. An examination of substance abuse treatment and Oregon statewide child welfare data reported reunification was more likely when parents obtained services soon after their child was placed out-of-home, spent more time in treatment and completed at least one treatment [27]. A study using Illinois child welfare data identified high rates of co-occurring problems among substance-using mothers and found that these problems, which included mental health, parenting, and employment related issues, decreased the likelihood of reunification [28]. There was a greater likelihood of reunification when services were matched to meet mothers' identified needs. A survey study of predominantly poor, urban, substance-using women with child welfare involvement found service matching on counseling services was associated with less substance use and ancillary service matching was associated with client satisfaction [29]. The total number of services received had the strongest impact on treatment outcomes.

### Vulnerable Infants Program of Rhode Island

The Vulnerable Infants Program of Rhode Island (VIP-RI) began as a demonstration grant to provide care coordination services to mothers with an open child welfare case because of drug use during pregnancy. Enrollment in VIP-RI typically occurs during a mother's hospitalization following delivery. When prenatal substance exposure is identified either through maternal self-report or a positive toxicology screen, a hospital social worker informs the mother about VIP-RI and with her consent, makes a referral to the program. The VIP-RI care coordinator assesses maternal and infant needs and facilitates referrals to appropriate services. VIP-RI remains involved with families until a permanent placement for the infant has been identified. In some instances, because of the voluntary nature of the program, families may withdraw before a decision regarding permanency has been made.

Mothers who participate in VIP-RI have the option of participating in the Rhode Island Family Treatment Drug Court (RI FTDC). RI FTDC is a specialized court for perinatal substance users that provides structure and support and takes an interactive, therapeutic approach that involves close monitoring and making referrals to substance abuse treatment and ancillary services.

VIP-RI's model of closely collaborating with social service agencies expedites parents obtaining the services and supports they need. Services typically arranged by VIP-RI include substance abuse treatment, mental health, medical care, parenting, and help obtaining entitlement benefits. Such services are generally accepted as helpful for perinatal substance users, however, little is known about which services are actually utilized and their relevance to child welfare outcomes. The purpose of this paper is to describe the services used by mothers who participated in VIP-RI and the following child welfare outcomes: 1) status of the child welfare case 2) reunification with biological mother and 3) an identified permanent placement for the infant. To our knowledge this is the first study to describe services for perinatal substance users and child welfare outcomes.

## Methods

An analysis of data from the first four years of VIP-RI identified services used by mothers while participating in VIP-RI and if the child welfare case had been closed, if reunification had been achieved, and if a permanent placement for the infant had been established. The hospital Institutional Review Board granted approval for the pilot VIP-RI program.

At enrollment, VIP-RI staff administered a semi-structured face-to-face psychosocial history interview that included measures of maternal characteristics and information about legal invovlement, substance abuse and treatment histories, trauma, and services received. The Substance Abuse Subtle Screening Inventory-3 [30] was used to screen for substance dependence, the Brief Symptom Inventory [31] identified mental health symptoms and the Adult - Adolescent Parenting Inventory - 2 [32] assessed high-risk parenting attitudes. Each of these standardized measures has established validity and reliability. Information obtained from the standardized measures and the psychosocial history interview were used as the basis for identifying services mothers needed. A major component of ongoing care coordination was monitoring compliance with service plans and keeping track of services VIP-RI participants recieived over the course of their involvement in the program.

Services were divided into eight categories shown in Table 1. Services were set as dichotomous yes/no variables to indicate if the service was received or not. The three child welfare outcomes examined were: 1) a closed child welfare case (yes/no) 2) reunification with biological mother (yes/no) and 3) an identified permanent placement for the infant (yes/no). Child welfare outcomes were obtained through court and VIP-RI records. The National Perinatal Information Center, an independent organization, monitored data collection and evaluated the impact of VIP-RI on permanency outcomes of substance-exposed infants.

**Table 1:** Services for VIP-RI Participants

Substance Abuse Treatment	Mental Health Treatment
Self-help	Mental health counseling/therapy
Outpatient drug treatment	Psychotropic medication management
Hospital-based treatment	Peer counseling
Residential drug treatment	
Residential facility for women & children	
Inpatient/outpatient detoxification	
	
Medical Care	Parenting/Child Care
HIV education/prevention	Child care
HIV screening/assessment	Parenting classes/training and/or support services
HIV services/treatment	Respite care
HIV pre/post test counseling	
Prenatal care	
Postnatal care	
Public health nurse visit	
Primary medical care	
Family planning	
	
Financial	Legal
Financial/entitlement assistance	Legal services/advocacy
Food/clothing donations	
Housing/rental assistance	
	
Education/Vocational	Other Services
Educational/schooling/GED assistance	Case management
Vocational/employment/job training	Domestic violence services
	In-home services
	Pastoral care
	Permanency services
	Recovery support services
	Transportation

### Data analysis

Chi-Square analysis was performed comparing dichotomous variables (services received, yes or no) and child welfare outcome variables (child welfare case closed, Y/N, was the infant reunified with the biological mother, Y/N, and was a permanent placement for the infant identified, Y/N).

#### Descriptive Results

### Maternal Demographics

During the first four years, 70% of mothers referred to VIP-RI enrolled, for a total of 195 mothers. Reasons for not enrolling were active or passive refusal (64%) (e.g., not interested in the program, lack of follow through after initial contact), ineligibility when a child welfare case was not opened after the initial CPS investigation (16%), and not being appropriate for reasons that included current incarceration, planning to place the child for adoption, psychiatric issues, not substance abuse, being identified as the primary problem (20%).

Most participants in VIP-RI were Caucasian (56%), followed by African American (22%), Hispanic (14%), and other (8%). Primary drugs of choice were cocaine (46%), opiates (27%), marijuana (24%) and alcohol (3%). Maternal ages ranged from 17 to 43 years (M= 28.4, SD = 6.03). At the time of enrollment in VIP-RI, 89% of the mothers were single and had an average of three children (range 1 - 9). Over one third of the sample had less than a high school education (37%), 61% had a high school diploma or equivalent, and 2% had a four-year college degree. Most mothers had no reliable source of income or received disability or entitlement benefits; only 6% were employed.

A higher proportion of mothers with at least a high school education (49.1% vs. 34.1%, p = 0.033), fewer children (1.53 vs. 1.63, p = 0.040), or only one child (27.0% vs. 15.0%, p = 0.036) had closed child welfare cases. Maternal characteristics associated with having an open child welfare case were childhood physical abuse (47.3% vs. 30.3%, p = 0.015) and a history of criminal conviction (40.4% vs. 25.6%, p = 0.030).

Similar results were found when maternal characteristics and reunification outcomes were analyzed. A greater proportion of mothers with fewer children (1.51 vs. 1.60, p = 0.040) or only one child (26.5% vs. 14.7%, p = 0.038) achieved reunification. A smaller proportion of mothers with less than a high school education were reunified with their children (31.7% vs. 51.5%, p = 0.004). A smaller proportion of mothers with a history of childhood physical abuse achieved reunification with their infants (31.7% vs. 49%, p = 0.012).

At the time of hospital discharge, 32% of infants remained with biological parents, 32% were placed in kinship care, 32% were placed in non-relative foster care and 4% went to specilaized care. Infant placements at the time of discharge from VIP-RI were 56% living with biological parents, 22% in kindship care and 22% in non-relative foster care. There were no statistically significant associations between maternal characteristics and child welfare outcome measures and maternal race, income or age. There also were no statistically significant associations between maternal characteristics and infant permanent placements.

### Findings Related to Child Welfare

#### Closed Child Welfare Cases

As shown in Table 2, HIV pre/post test counseling, prenatal and postnatal care, primary medical care, family planning, entitlement assistance and donations of food and clothing, were the services associated with a greater percentage of closed child welfare cases.

**Table 2:** Child Welfare Status: Closed Case

	N	Closed Child Welfare Case	N	Open Child Welfare Case	p
Total Cases	89		113		
Medical Care Services					
HIV screening/assessment	77	28(36.4%)	95	20(21.1%)	< 0.001
HIV pre/post test counseling	77	33(42.9%)	95	13(13.7%)	< 0.001
Prenatal care	85	61(71.8%)	108	46(42.6%)	<.001
Postnatal care	87	75(86.2%)	111	61(55%)	<.001
Public health nurse visit	86	32(37.2)	106	24(22.6%)	0.027
Primary medical care	87	74(85.1%)	108	57(52.8%)	<.001
Family planning	81	63(77.8%)	102	45(44.1%)	<.001
Financial Services					
Entitlement assistance	85	65(76.5%)	110	44(40%)	<.001
Food/clothing donations	85	56(65.9%)	110	42(38.2%)	<.001
Housing/Rental Assistance	85	38(44.7%)	109	31(28.4%)	0.019
Educational/Vocational Services					
Educational/Schooling/GED	85	18(21.2%)	111	9( 8.1%)	0.009
Mental Health Treatment					
Peer Counseling	86	35(40.7%)	107	25(23.4%)	0.010
Parenting/Child Care					
Child Care	85	37(43.5%)	110	25(22.7%)	0.002

#### Reunification

As shown in Table 3, HIV pre/post test counseling, primary medical care, family planning, entitlement assistance and donations of food and clothing, and recovery support services were associated with a greater percentage of reunification with the biological mother. Residential drug treatment and permanency services were less likely to be associated with reunification with the biological mother.

**Table 3:** Child Welfare Status: Reunification

	N	Not Reunified with Biological Mother	N	Reunified with Biological Mother	p
Total Cases	102		102		
Medical Care Services					
HIV pre/post test counseling	87	13(14.9%)	86	31(36.0%)	0.001
Primary medical care	97	56(57.7%)	99	73(73.7%)	0.018
Family planning	87	42(48.3%)	97	65(67%)	0.010
Financial Services					
Entitlement assistance	99	43(43.4%)	99	69(69.7%)	<.001
Food/clothing donations	98	42(42.9%)	99	59(59.6%)	0.019
In-home Services	100	53(53.0%)	99	71(71.7%)	0.006
Substance Abuse Treatment					
Residential Drug Treatment	98	39(39.8%)	99	20(20.2%)	0.003
Other Services					
Permanency services	97	60(61.9%)	99	40(40.4%)	0.003
Recovery support services	96	77(80.2)	99	92(92.9%)	0.009

#### Permanent Placement

As shown in Table 4, HIV pre/post test counseling and recovery support services were associated with a greater proportion of infants with permanent placements.

**Table 4:** Child Welfare Status: Permanent Placement

	N	Permanent Placement Not Identified	N	Permanent Placement Identified	p
Total Cases	73		131		
Medical Care Services					
HIV pre/post test counseling	59	4( 6.8%)	114	40(35.1%)	<.001
Other Services					
Permanency Services	71	44(62.0%)	125	56(44.8%)	0.021
Recovery Support Services	69	55(79.7%)	126	114(90.5%)	0.034

## Discussion

Families with parental substance use and child welfare involvement are less likely to reunify and more likely to experience lengthy stays in foster care and higher rates of re-reporting [27,33-36]. Increasing our understanding of which services are associated with better child welfare outcomes has applicability to the child welfare system, court, treatment providers, and to families. Results of this study can be used to generate hypotheses about how to prioritize services in a time of diminishing resources.

In this study, the majority of services related to closed child welfare cases and reunification with biological mother were medical and financial services. Medical services included HIV pre/post test counseling, prenatal and postnatal care, primary medical care and family planning. Medical professionals should be aware of the positive impact they can have on the lives of substance-exposed infants and parents beyond the medical attention they provide. A strong connection with medical providers can serve families well in terms of following through with routine medical visits and providing a foundation of care for a mother to address concerns about her own and her children's health.

Financial services that were associated with positive child welfare outcomes included assistance with entitlement benefits and obtaining food and clothing. Stress associated with not having essential needs met can interfere with a woman's ability to focus on treatment and may contribute to her questioning if she is able to adequately provide for her children. Ensuring that a family's basic needs are met may have the additional benefits of promoting a mother's ability to work on recovery and child welfare case plans by allaying worries about how to feed, clothe and shelter her children. Building in these services as part of child welfare, treatment, or court may contribute to more positive child welfare outcomes.

Not all services were associated with favorable child welfare outcomes. Permanency services and residential drug treatment were less likely to be associated with reunification with the biological mother or identified permanent placements for infants. Permanency services included providing support to families who were voluntarily or involuntarily having parental rights terminated, which may account for these services being associated with infants not being reunified or having established permanent placements. Residential treatment may have been a marker for more severe addiction and related risk factors, which may have indicated a poorer prognosis.

### Study Limitations

Involvement in child welfare because of drug use during pregnancy is a complicated issue and numerous factors affect child welfare outcomes. This study did not control for other factors that could have influenced child welfare outcome, such as social support networks, satisfaction with services, etc. This study did not correct for multiple comparisons because the purpose of the study was to describe services related to specific outcomes, as this had not been done previously. Adjustment for multiple comparisons protects against rejecting the null hypothesis when it is correct (type I error). However, the cost of this protection is to increase type II error that findings are attributable to chance when they are not.

In addition, this study is limited to a population of perinatal substance users who participated in an innovative program, VIP-RI, which has an active partnership with the state's FTDC. There was no comparison group of mothers with open child welfare cases because of drug use during pregnancy who did not participate in VIP-RI.

## Conclusion

Services that addressed family health and financial needs were associated with favorable child welfare outcomes for perinatal substance users with child welfare involvement. When considering the range of services substance-using women often need, it is important to make sure that their basic needs are not overlooked. Alleviating health and financial pressures may have positive consequences for women struggling to address substance abuse while attempting to assume parenting responsibilities by allowing them to focus more on their recovery and parenting skills as they attempt to succeed with their child welfare case plans.

## Competing interests

The authors declare that they have no competing interests.

## Authors' contributions

KJM, JET & BML conceived of the study and wrote the manuscript. DC performed the statistical analysis. RS & LA coordinated VIP-RI and the collection of data. All authors have read and approved the final manuscript.
